# Daily physical activity and related risk factors in COPD

**DOI:** 10.1186/s12890-020-1097-y

**Published:** 2020-03-05

**Authors:** Ali M. Albarrati, Nichola S. Gale, Maggie M. Munnery, John R. Cockcroft, Dennis J. Shale

**Affiliations:** 1 School of Healthcare Sciences, University Hospital of Wales, Cardiff University, Cardiff, UK; 2 Rehabilitation Sciences Department, College of Applied Medical Sciences, King Saud University, P.O Box 10219, Riyadh, 11433 Kingdom of Saudi Arabia; 3 Cardio-Respiratory Medicine, Wales Heart Research Institute, Cardiff University, University Hospital of Wales, Heath Park Campus, Cardiff, CF14 4XN UK

**Keywords:** BODE index, Breathlessness, Daily activity, Exacerbation, Handgrip strength

## Abstract

**Background:**

Factors associated with reduced daily physical activity (DPA) in patients with COPD are still controversial. Physical inactivity in COPD increases risk of cardiovascular disease, frequent exacerbations, reduced health status, and increased symptoms. We hypothesised that reduced DPA in patients with COPD is independent of traditional risk factors including age and spirometry.

**Methods:**

In this cross-sectional study, DPA (over 7 days) was assessed on 88 community stable patients with COPD and 40 controls free from cardiorespiratory disease. Spirometry, body composition, number of exacerbations, handgrip strength (HGS), modified Medical Research Council (mMRC), arterial stiffness, 6-min walking distance (6MWD) and BODE index were also determined. Frequent exacerbation was defined as ≥2 and non-frequent exacerbation < 2.

**Results:**

Patients with COPD had reduced DPA and exercise capacity compared with controls similar in age, BMI and gender, *p* < 0.001. Frequent exacerbators had less DPA than infrequent exacerbators and both less than controls, p < 0.001. Patients with higher BODE index were less active than those with lower index. Time spent on moderate activity was related to cardiovascular risk factors including arterial stiffness. The DPA in patients was independent of age, gender, spirometry, body composition and HGS, *p* > 0.05. The level of breathlessness was superior to lung function in predicting the level of DPA.

**Conclusion:**

The level of DPA in COPD was independent of traditional risk factors. Breathlessness score is a better predictor of the DPA than lung function and handgrip strength.

## Background

Reduced daily physical activity (DPA) is a multifactorial deficit in chronic obstructive pulmonary disease [1]. Reduced DPA is a key contributor to cardiovascular morbidity, recurrent hospital admissions related to exacerbations, disease progression and reduced health related quality of life [2]. Physical inactivity is also the strongest predictor for all-cause mortality in this population [3]. Nevertheless, it is modifiable and studies indicate that engaging in a regular physical activity could reduce the risk of cardiovascular disease, hospitalisation rate and death related to COPD [4, 5]. Garcia-Aymerich and colleagues found that participation in physical activity equivalent to walking or cycling for at least 2 h a week was associated with a 30–40% reduction in the risk of COPD related hospital admission and mortality [5].

A number of studies have tried to identify factors related to declining of physical activity in COPD and yielded controversial results [6–8]. Most common risk factors reported in these studies are reduced lung function, musculoskeletal strength, breathlessness score and inflammation. The discrepancies in the results may be attributed to the methodological variations as the patients recruited in these studies were either participating in an inpatient or outpatient rehabilitation program which may not represent typical patients with COPD. Patients with COPD are usually referred to rehabilitation program when they report significant breathlessness and may be physically inactive and deconditioned. This may not reflect the general, less symptomatic COPD population, who may remain active and have a reasonable health status. Moreover, the control subjects recruited in these studies were relatively active and may not be representative of the older population.

Therefore, we hypothesised that decreased DPA in stable community patients with COPD would be independent of traditional risk factors.

## Methods

### Subjects

This is a cross sectional analysis from a large prospective longitudinal study in COPD (The ARCADE study, Clinical Trials No NCT 01656421) [9]. A convenience sample of 93 patients with COPD confirmed with post bronchodilator spirometry and 42 controls, either current or ex-smokers free from respiratory disease were recruited.

All patients were clinically stable and had not had any oral corticosteroids or antibiotics 4 weeks prior to recruitment. Patients were excluded if they were known with inflammatory disease such as rheumatoid arthritis, had oral maintenance corticosteroids and long-term oxygen therapy or had attended pulmonary rehabilitation. Participants were recruited from respiratory outpatient clinics, smoking cessation referrals and general practice databases. Controls were recruited from general practice databases, smoking cessation clinic and patients’ relatives. All participants gave written informed consent and the study had approval from the South East Wales Research Ethics Committee.

### Anthropometry and body composition measurement

In all participants, height and weight was measured barefoot with subjects wearing lightweight clothing and body composition was assesed using a single frequency segmental bioelectrical impedance analyser (BC-418 MA, Tanita Corp., Tokyo, Japan). Body mass index (BMI kg/m^2^), the fat free mass and fat mass were also determined. Waist and hip circumference were measured with a stretch resistant tape [10].

### Lung function and COPD related questionnaires

All participants performed spirometry (Vitalograph alpha, Bucks, UK) to measure the forced expiratory volume in 1 s (FEV_1_), the forced vital capacity (FVC) and FEV_1_/FVC ratio. Patients were given 400 μg of salbutamol via a spacer device and repeated the test 10 min post bronchodilator. Patients were asked to abstain using their inhalers for at least 6 h prior to their visit [11] .

Breathlessness was scored using the modified Medical Research Council (MRC) dyspnoea scale. The number of exacerbations in the last year (defined as requirement for antibiotic or oral corticosteroid therapy per year) were recorded. Patients and controls reported also the number of previously diagnosed comorbidities. Infrequent exacerbators were defined as < 2 in the previous year, while frequent exacerbators as ≥2 [12].

Patients completed the St George’s Respiratory Questionnaire (SGRQ) and the COPD assessment test (CAT), both validated measures of health status and symptoms [13, 14].

### Daily physical activity assessment

Participants were given a physical activity monitor (SenseWear™ BodyMedia, Inc. Pittsburgh, PA) on the day of their visit to Wales Heart Research Institute and asked to return the monitor after a week from their visit. The first and last days were excluded from the analysis because of incomplete measurements of the days. Therefore, data from 6 days (4 weekdays plus the weekend) of measurements were available for most participants. Participants were asked to wear the monitor the whole day except during shower or sleep. Wearing time was recorded by the monitor. For a valid day of activity measurement, the threshold was set at 16 h of monitor’s wearing time. Days below that threshold were excluded from analysis. The monitor has been found to be reliable and valid in patients with COPD [15]. To avoid seasonal changes, data were collected from the beginning of May until the end of September. The variables chosen for this analysis were the total daily number of steps (Steps) and daily time spent in at least moderate physical activity as defined by any activity ≥3 METs. Step count was divided to above or below 5000 steps/day as a cut-off value of recommended steps per day [16].

### Physical performance

The 6MWD was performed once according to the American Thoracic Society (ATS) guideline using a 30-m level, straight indoor track [17]. Maximal handgrip strength (HGS) was determined twice in each hand and the mean was calculated for each one using a hand dynamometer (T.K.K. 5401 grip-D, Takei, Japan).

### BODE index

BODE index score was calculated by adding the score of each variable (BMI, post bronchodilator FEV_1_, mMRC and 6MWD).

### Aortic pulse wave velocity

Peripheral systolic and diastolic BP was measured after 10 min rest in seated and supine positions (OMRON Corporation, Kyoto, Japan). Aortic PWV and central blood pressures including mean arterial pressure (MAP) measured noninvasively using SphygmoCor device (AtCor Medical, Sydney, Australia) [18].

### Inflammatory biomarkers

High sensitivity C – reactive protein (HsCRP) and fibrinogen were determined by standard assays (Biochemistry, University Hospital of Wales).

### Statistical analysis

All statistical analysis was performed using the statistical software package 23.0 (Chicago, Illinois, U.S.A.). Data were checked for normality prior to analysis. Data were presented as mean and standard deviation or median and interquartile range (IQR). Between groups comparisons were performed using analysis of variance. Categorical data was analysed using the Chi-square test. Correlations between variables were explored using Pearson’s and Spearman correlation coefficients. Multivariate analysis was performed using a stepwise multiple regression model. For all analysis *p* < 0.05 was considered significant.

## Results

**Fig. 1:**
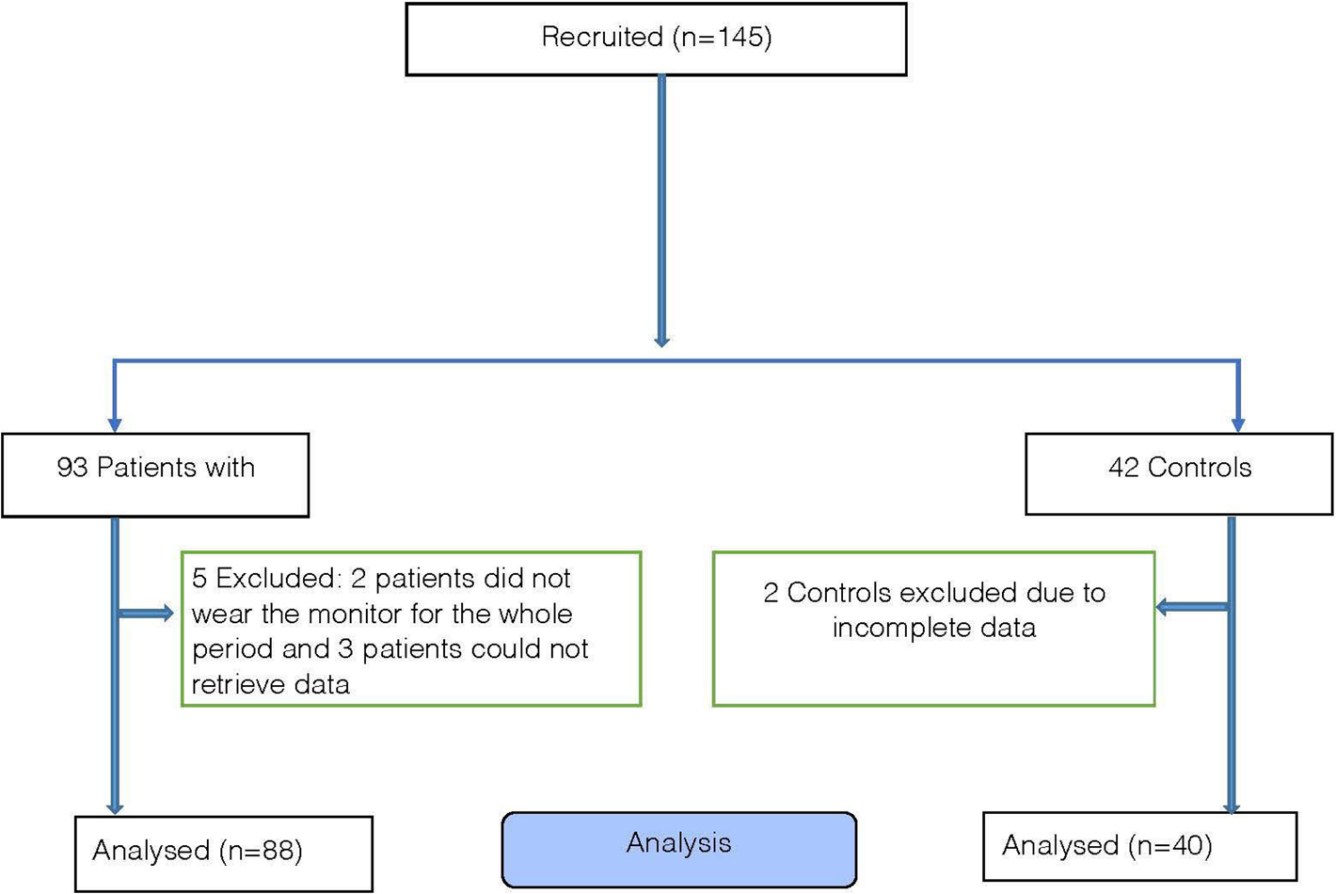
Consort Diagram

Eighty-eight patients and forty controls had complete data (Fig. 1). Five patients and two controls were removed from the analysis. Two patients and two controls did not wear the monitor for seven days and therefore were excluded. Three patients returned the monitor after 14 days and the batteries of the monitors were dead and we could not retrieve the data after charging the monitors. The patients and controls were similar in age, gender proportion and BMI. The patients had poorer lung function (FEV_1_, FVC and their ratio), lower resting oxygen saturations and greater tobacco exposure than the control group (all *p* < 0.001) (Table 1). The severity of airflow obstruction by GOLD stratification (Table 2). The distribution of the modified MRC breathlessness score was: mMRC 0 *n* = 11, mMRC 1 *n* = 34, mMRC 2 = 17, mMRC 3 *n* = 18 and mMRC 4 *n* = 8. The patients were also classified according to GOLD quadrants based on CAT score (Table 2).

**Table 1:** Physical and Clinical Characteristics of patients with COPD and Controls

	COPD (*n* = 88)	Comparator (*n* = 40)	*p*
Gender Male: female	46:42	19:21	0.117
Age (years)	66 (8)	66 (7)	0.923
Smoking (pack years)	42.2 (24.8)	22.9 (20.8)	0.001
FEV_1_ (L)	1.5 (0.8)	2.6 (0.6)	0.001
FVC (L)	2.4 (0.9)	3.4 (0.9)	0.001
FEV_1_/FVC	0.52 (0.1)	0.78 (0.05)	0.001
FEV_1_ (%)	56 (17)	106 (17)	0.001
FVC (%)	84 (19)	112 (17)	0.001
Resting O_2_ saturation (%)	96 (2)	98 (1)	0.001
BMI (kg/m^2^)	27.5 (5.2)	28.6 (4.9)	0.297
Waist:Hip ratio	0.95 (0.09)	0.89 (0.06)	0.002
FFM (kg)	49.5 (10.5)	50.9 (9.1)	0.486
FM (kg)	24.8 (9.9)	27.2 (10.3)	0.243
FFM/FM	2.3 (1.1)	2.2 (1.1)	0.556
FFMI (kg/m^2^)	18.2 (2.4)	18.5 (2.1)	0.589
FMI (kg/m^2^)	9.3 (3.9)	10.1 (4.3)	0.3
6MWD (m)	336 (107)	515 (88)	0.001
Mean handgrip (kg)	27.2 (9.6)	29.7 (10.2)	0.209
Fibrinogen (g/l) #	3.8 (1.3)	3.2 (0.6)	0.012
HsCRP (mg/l)#	3.3 (3.1)	1.6 (3.2)	0.023
No. Steps	4095 (2720)	6734 (3491)	0.001
Time spent on moderate activity (hr)*	1.3 (0.5–2.2)	1.3 (0.7–2.1)	0.662
No. Comorbidity	1.3 (1.2)	0.8 (0.8)	0.027

All data mean (SD) unless otherwise indicated *Median (range) # Geometric mean. *Abbreviations*: *6MWD* six minute walk distance, *BMI* body mass index, *FEV1* forced expiratory volume in 1 s, *FVC* forced vital capacity, *FFM* fat free mass, *FFMI* fat free mass index, *FM* fat mass, *FMI* fat mass index, *HsCRP* high sensitivity C- reactive protein.

**Table 2:** Distribution of Patients with COPD according to GOLD classifications and Quadrants

GOLD Classification	Patients Number	GOLD Quadrants	Patients Number
GOLD I	9	GOLD A	8
GOLD II	50	GOLD B	24
GOLD III	25	GOLD C	10
GOLD IV	4	GOLD D	46

### Measures of daily physical activity

The DPA was lower in the patients, mean (SD), 4095 (2720) steps than controls 6734 (3491) steps, *p* < 0.001, however, there was no difference in DPA for males and females in either group, *p* > 0.05. The DPA was not related to age in either patients or controls. Using the minimum recommended level of DPA of 5000 steps/day, only 31% of the patients had reached the recommended amount of physical activity; while in contrast, 73% of the controls met the minimum amount of the DPA.

The DPA related to FEV_1_% predicted (r = 0.39, *p* < 0.001), oxygen saturation (*r*_*s*_ = 0.29, *p* = 0.012) and mMRC dyspnoea score (r = −0.45, *p* < 0.001), in the patients. Only was FEV_1_% predicted related to DPA in the controls, r = 0.39, *p* = 0.017.

### Daily physical activity and disease severity

**Fig. 2:**
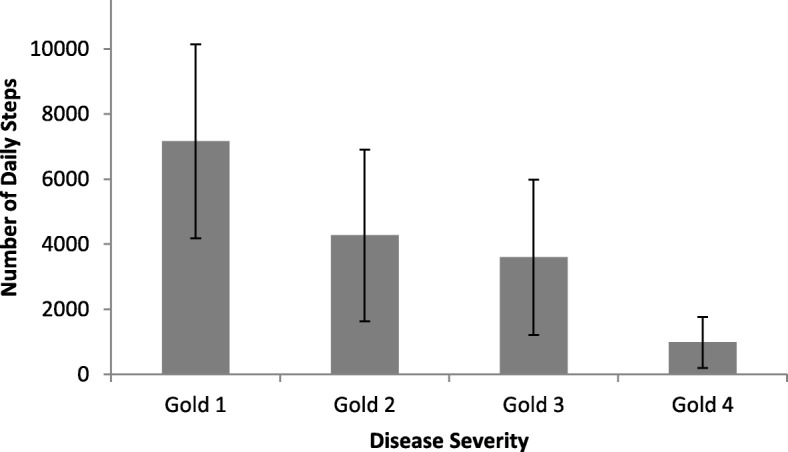
Daily physical activity disease severity (GOLD categories). Bars represent means and vertical lines represent upper and lower 95% confidence intervals for each category

**Fig. 3:**
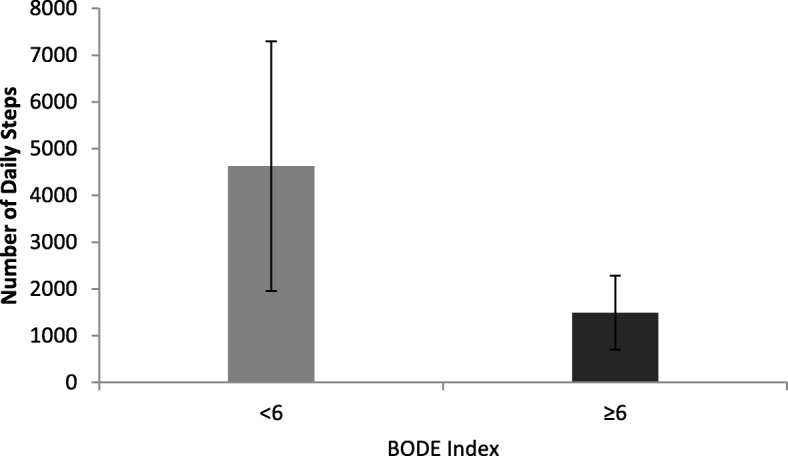
Daily Physical activity and BODE index<or > 6. Bars represent means and vertical lines represent upper and lower 95% confidence intervals for each category

Level of DPA differed across the GOLD categories, between GOLD 1 and GOLD 4, *p* = 0.004 (Fig. 2). Patients with an FEV_1_ > 50% had greater level of DPA, lower exacerbation rate, breathlessness score and CAT score than patients with an FEV_1_ < 50%, *p* < 0.001. There was an association between level of DPA and BODE index, *p* < 0.001. Patients with BODE score of ≥6 walked less significantly than patients with a lower score, *p* < 0.001 (Fig. 3).

### Daily physical activity and body composition and physical function

There was no relationship between body composition parameters and DPA in the patients or control group. However, in the patients, the time spent undertaking moderate activity was related to BMI, r = − 0.39, FM, r = − 0.37, FFM:FM, r = 0.50, all *p* < 0.001. Similar relationships were evident in the control group, all *p* < 0.05.

The 6MWD and HGS were less in patients than controls, both *p* < 0.001 (Table 1). In the patients, the level of daily of daily physical activity was related to the 6MWD, r = 0.45, *p* = 0.001, and controls, r = 0.49, *p* = 0.002, but it was not related to HGS, *p* > 0.05. The 6MWD and HGS were all related to each other in the patients, *p* < 0.01.

### Daily physical activity and aortic stiffness

In the patients, the time spent undertaking moderate activity was related to aortic stiffness (*rs* = − 0.28, *p* < 0.01), resting oxygen saturation (*rs* = − 0.38, *p* < 0.002). This was not evident in controls. Aortic stiffness was greater in patients, 9.5 (2.2), than controls, 8.2 (1.4),*p* < 0.01.

### Patient reported outcomes

The level of DPA and the SGRQ total score were related (r = − 0.38, *p* < 0.001), as was the SGRQ activity domain (r = − 0.40, *p* < 0.001). The CAT score was also related to the level DPA (r = − 0.34, *p* = 0.003). The 6MWD also related to the total SGRQ score, r = − 0.63, and the CAT score r = − 0.54, (all *p* < 0.001).

Of the patients, 41 reported 0–1 exacerbations/ year and 47 reported two or more exacerbations/ year. The level of DPA was related to the frequency of exacerbations r = − 0.34, *p* < 0.001, and frequent exacerbators, 3348 (2221) steps, had lower physical activity of daily living than infrequent exacerbators, 5094 (3027) steps.

### Systemic inflammation

Circulating CRP and fibrinogen were greater in patients than controls (*p* < 0.001) and both were related to the level of DPA, CRP, *r*_*s*_ = − 0.29, *p* = 0.013, and fibrinogen, r = − 0.30, *p* = 0.009, but were unrelated to the level of DPA in the control group.

### Predictive factors for the daily physical activity

In the patients, a stepwise multivariate regression analysis including DPA as a dependent variable and FEV_1_%, mMRC and the number of exacerbations as the independent variables showed that mMRC and number of exacerbations explained 28% of the variability in the DPA with FEV_1_% predicted excluded from the analysis, adjusted R^2^ = 0.28,*p* < 0.001. The mMRC and the number of exacerbations explained 34 and 25% of the reduction in the DPA, respectively.

## Discussion

To our knowledge, this is the first study to comprehensively examine risk factors associated with daily physical activity in stable community patients with COPD and controls similar in age and gender. The DPA in COPD was associated with level of breathlessness and frequency of exacerbations, independent of age, lung function, sex, peripheral muscle mass strength.

The GOLD classification was not discriminative enough between patients with moderate and severe impaired DPA. The level of breathlessness was found to be superior to lung function. This may be explained as a number of studies have found that FEV_1_ is less sensitive to describe the complexity of COPD and weakly related to the level of breathlessness [19, 20]. Breathlessness is the most common reason for visiting primary care and respiratory physicians regardless the severity of lung function [21]. A recent study on patients attending primary care found physical inactivity was associated with breathlessness independent of lung function [22]. Perception of breathlessness, which patients experience during performing DPA, is involved in a complex mechanism beyond respiratory mechanical factors [23]. Patients with severe breathlessness often limit their DPA to minimise dyspnoea and creating a vicious cycle of inactivity, muscle atrophy, deconditioning and which result in greater activity intolerance [15, 24–27]. Similar findings reported by other researchers confirming the independent relationship between disease severity and DPA [8, 28].

Patients with poor prognosis (BODE ≥6) had limited DPA which is a strong predictor of poor outcomes including mortality [29, 30]. Similarly, a number of studies reported in a small cohort study in patients with COPD a direct relationship between physical inactivity and increased BODE index [24, 31]. This is supported by the concept that reduced DPA is a strong predictor of morbidity and mortality in patients with COPD [3, 5]. Pitta and colleagues found inactivity for just one day doubled the BODE score, which increased the probability of premature mortality [24]. BODE index is a multidimensional and appears to have the capacity to stratify patients better than a single marker of airway severity, as FEV_1_ cannot reflects the whole picture of COPD.

Reduced DPA increases the risk of cardiovascular disease and its related risk factors including arterial stiffness. Arterial stiffness is a validated marker of CV morbidity and mortality. A few studies have examined the relationship between DPA and arterial stiffness in COPD and showed different results [32, 33]. In our study, moderate DPA was associated with reduced arterial stiffness. Increased arterial stiffness is a surrogate marker of increased risk of cardiovascular disease in COPD, which could be reduced by increasing DPA [34–37]. Indeed, a number of studies found a reduction in aortic stiffness after patients with COPD attended a structured programme of pulmonary rehabilitation [37, 38]. A recent study found that patients engaging in any type of moderate physical activity was associated with lower mortality rate after one year compared to inactive patients [39].

Altered body composition is an accepted feature of COPD with several studies reporting reduced musculoskeletal mass and strength [40–43]. However, in our study, neither muscle mass nor HGS was associated with reduced DPA and exercise capacity. Van Gestel and colleagues reported a similar finding to our study and showed that HGS was not associated with DPA and could not be used to predict DPA [44]. A possible explanation is that despite the limitations of lower limb activities by breathlessness, upper limb activities and strength may be maintained [1].

In addition, the larger lower limb mass may be more affected by circulating inflammation, a key feature of COPD. Increased circulatory inflammation may profoundly alter musculoskeletal function, increase muscle oxidative stress and accelerate muscle protein degradation [21, 45]. Systemic inflammation (CRP) was elevated in COPD in the present study and was associated with fat mass a potential source of inflammation and impaired DPA. Increased inflammatory biomarkers i.e. CRP and fibrinogen, which are associated with increased risk of cardiovascular disease and insulin resistance, and their association with reduced DPA has been previously shown in individuals with COPD and community dwellers [46, 47]. Increased systemic inflammation (SI) is linked to an exacerbation frequency, which is associated with breathlessness and impaired DPA [46].

### Limitations

A key limitation of this study is its cross-sectional nature, and this issue is being addressed in the ongoing longitudinal ARCADE study. We had a limited number of DPA monitors so which curtailed recruitment.

## Conclusion

The increased level of breathlessness and frequency of exacerbation are key factors in limiting daily physical activity in patients with COPD independent of loss of muscle mass and strength. The association between reduced DPA and breathlessness suggests that interventions to improve perception of breathlessness may optimise patient outcomes including physical activity. There is a need for long-term behavioural interventions to increase/maintain DPA in patients with COPD.

## Data Availability

The datasets generated and/or analysed during the current study are not publicly available due other manuscripts will be published from this data, but are available from the corresponding author on reasonable request.

## Abbreviations

6MWD: Six minute walk distance
BMI: Body mass index
DPA: Daily physical activity
FEV1: Forced expiratory volume in 1 s
FFM: Fat free mass
FFMI: Fat free mass index
FM: Fat mass
FMI: Fat mass index
FVC: Forced vital capacity
HGS: Handgrip strength
HsCRP: High sensitivity C- reactive protein
SI: Systemic inflammation
